# Comparative Analysis of HMC3 and C20 Microglial Cell Lines Reveals Differential Myeloid Characteristics and Responses to Immune Stimuli

**DOI:** 10.1111/imm.13900

**Published:** 2025-02-17

**Authors:** Bavani Gunasegaran, Shivani Krishnamurthy, Sharron S. Chow, Millijoy D. Villanueva, Anna Guller, Seong Beom Ahn, Benjamin Heng

**Affiliations:** ^1^ Macquarie Medical School, Faculty of Medicine, Health and Human Sciences Macquarie University Sydney New South Wales Australia; ^2^ Computational Neurosurgery (CNS) Lab, Macquarie Medical School, Faculty of Medicine, Health and Human Sciences Macquarie University Sydney New South Wales Australia

**Keywords:** immune, infection: Myeloid, inflammation, microglia

## Abstract

Microglia are the primary resident immune cells of the central nervous system (CNS) that respond to injury and infections. Being critical to CNS homeostasis, microglia also have been shown to contribute to neurodegenerative diseases and brain cancer. Hence, microglia are regarded as a potential therapeutic target in CNS diseases, resulting in an increased demand for reliable in vitro models. Two human microglia cell lines (HMC3 and C20) are being used in multiple in vitro studies, however, the knowledge of their biological and immunological characteristics remains limited. Our aim was to identify and compare the biological changes in these immortalised immune cells under normal physiological and immunologically challenged conditions. Using high‐resolution quantitative mass spectrometry, we have examined in‐depth proteomic profiles of non‐stimulated and LPS or IFN‐γ challenged HMC3 and C20 cells. Our findings reveal that HMC3 cells responded to both treatments through upregulation of immune, metabolic, and antiviral pathways, while C20 cells showed a response associated with mitochondrial and immune activities. Additionally, the secretome analysis demonstrated that both cell lines release IL‐6 in response to LPS, while IFN‐γ treatment resulted in altered kynurenine pathway activity, highlighting distinct immune and metabolic adaptations.

## Graphical abstract

This study highlights comparative proteomic and morphological analyses of HMC3 and C20 microglial cell lines under baseline and inflammatory stimuli (LPS and IFN‐γ). Despite a 76.6% similarity in proteins, unstimulated HMC3 showed a transcriptional cell protein profile, whereas unstimulated C20 had a cell protein profile associated with the immune‐related pathway. Interferon‐γ treated HMC3 exhibited a cell protein profile linked to the immune response, while C20 had a cell protein profile associated with neuroinflammation and immune‐related activities. Both cell lines had minimal to no significant effect from lipopolysaccharide.

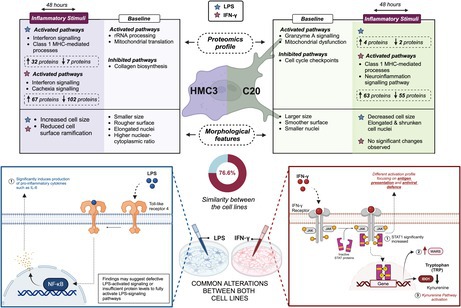

**Abbreviations**
ALDH4A1: Aldehyde Dehydrogenase 4 Family Member A1
ATP6V1G1: ATPase H+ Transporting V1 Subunit G1
BP: Biological process
CC: Cellular component
CNS: Central nervous system
CYP51A1: Cytochrome P450 Family 51 Subfamily A Member 1
DDA: Data‐dependent acquisition
DEPs: Differentially expressed proteins
DIA: Data‐independent acquisition
DNA: Deoxyribonucleic acid
EMG1: EMG1 N1‐Specific Pseudouridine Methyltransferase
FC: Fold change
FDR: False discovery rate
GNB2: G Protein Subunit Beta 2
GO: Gene Ontology
GSPT2: G1 To S Phase Transition 2
HCD: Higher‐energy collisional dissociation
HMC3: Human microglial clone 3
HPLC: High performance liquid chromatography
IDO1: Indoleamine 2,3‐dioxygenase‐1
IFN‐γ: Interferon‐gamma
IL: Interleukin
IPA: Ingenuity Pathway Analysis
ISG20: Interferon Stimulated Exonuclease Gene 20
ITGB3: Integrin Beta 3
JAK‐STAT: Janus Kinases‐Signal Transducer and Activator of Transcription
KRT10: Keratin 10
LPS: Lipopolysaccharide
MF: Molecular function
MVK: Mevalonate Kinase
NAMPT: Nicotinamide Phosphoribosyltransferase
NCR: Nuclear‐cytoplasmic ratio
NFYA: Nuclear Transcription Factor Y Subunit Alpha
NF‐κB: Nuclear Factor‐Kappa B
NIT1: Nitrilase 1
OSGEPL1: O‐Sialoglycoprotein Endopeptidase Like 1
PBS: Phosphate‐buffered saline
RNA: Ribonucleic acid
SDHAF2: Succinate Dehydrogenase Complex Assembly Factor 2
TIMM9: Translocase Of Inner Mitochondrial Membrane 9
UHPLC: Ultra‐high performance liquid chromatography
WARS1: Tryptophanyl‐TRNA Synthetase 1

## 1 Introduction

Microglia are resident macrophages [1] that reside within the parenchyma of the central nervous system (CNS) and are derived from myeloid progenitors in the yolk sac [2]. As the resident immune cell of the CNS, microglia are integral to the maintenance of CNS homeostasis [3] by responding to changes in its surrounding environment [4] and stimuli [5] by migrating to and resolving injuries and maintaining neuronal integrity [5, 6]. These environmental changes include the presence of damage‐associated molecular patterns and/or pathogen‐associated molecular patterns [7, 8] that activate the microglia to produce and release of cytokines and chemokines. These chemokines recruit additional immune cells, which then participate in protecting the CNS by resolving infections, inflammation [5, 7], or even early‐stage brain cancer [9, 10, 11]. Despite its primary neuroprotective role, sustained inflammation can cause microglia to remain reactive, contributing to a chronic neuroinflammatory state and leading to neurotoxicity [12].

In addition to the chronic production of inflammatory cytokines, inflammation can also upregulate the kynurenine pathway, a major biochemical pathway to catabolise the essential amino acid tryptophan to produce energy, in microglia [13]. This pathway is dysregulated predominantly in microglia within CNS in neurodegenerative diseases [14, 15]. Activation of this pathway not only depletes local tryptophan but also produces neurotoxic metabolites that lead to neuronal death [16]. Collectively, microglia exhibit both protective and detrimental roles in various neurological disorders [17] and cancer [18], which frequently comprise disease exacerbation via activation of inflammatory responses [19]. Hence, microglia have become one of the treatment targets in neurological diseases.

The difficulties in obtaining sufficient amounts of human brain microglia for research purposes and differences between human and animal myeloid cells have stimulated the studies on the development and characterisation of the permanent human microglial cell lines [20, 21, 22, 23] that could be invaluable tools for the analysis of the microglial behaviour in controlled laboratory settings.

Our study is specifically focused on two human microglia‐derived cell lines, human microglial clone 3 cell line (HMC3) [24] and C20. HMC3 originates from human foetal microglial cells [24, 25] and modified via SV40‐dependent immortalization [26]. It is often utilised in neuroinflammation research and drug screening [24, 27, 28, 29, 30]. It also serves as a model to investigate microglial activation and behaviour in response to various stimuli [23, 31]. In contrast, C20 was derived from adult human microglia via transduction with the combination of large SV40 T antigen and hTERT [32]. C20 finds its primary application in neuroinflammatory conditions [33, 34, 35, 36, 37].

Despite increasing use of HMC3 and C20 cell lines as in vitro models of human microglia, the current knowledge of their molecular characteristics remains limited. To better understand the alterations of biological/signalling pathways in HMC3 and C20 cells and their relevance to human CNS innate immunity, we carried out a comprehensive quantitative proteomic profiling of these cells treated with either lipopolysaccharide (LPS) or interferon‐gamma (IFN‐γ), commonly used to model neuroinflammatory reactions in microglia in vitro [38]. Additionally, the secretome of the untreated and LPS‐ or IFN‐γ‐challenged HMC3 and C20 cells was examined to understand its immunomodulating responses by measuring cytokines and chemokines as well as key metabolites of the kynurenine pathway. The results reported in the current study provide important insights for the in vitro modelling of human microglia and contribute to the overall knowledge of microglial function, enabling a rational selection of the testbeds for neuroinflammation, neurodegeneration and brain cancer research.

## 2 Methods

### 2.1 Cell Culture

HMC3 cells (CRL‐3304) were purchased from ATCC, and C20 cells were kindly donated by Prof. Alvarez‐Carbonell [32]. HMC3 and C20 cells were cultured with EMEM (30–2003, ATCC) and DMEM/F12 (Thermo Fisher Scientific, MA, USA) media, respectively, supplemented with 10% foetal bovine serum (FBS, Scientifix), 1% penicillin/streptomycin (Sigma‐Aldrich, MO, USA), and maintained at 37°C in a humidified atmosphere with 5% CO_2_.

### 2.2 Immune Challenge Treatment

Cells were seeded at 1 × 10^6^ cell density in 2 mL of cell culture medium in 6‐well plates. After 24 h, the culture medium was replaced and the cells were treated for 24 and 48 h with IFN‐γ (50 ng/mL) (Miltenyi Biotec), or LPS (1 μg/mL) (Sigma‐Aldrich, MO, USA). Untreated cells with media only were used as control, while phosphate‐buffered saline (PBS) (Thermo Fisher Scientific, MA, USA) was used as vehicle control for IFN‐γ and LPS treatments. Upon treatment, cell culture supernatant was collected at both 24 and 48 h for the kynurenine pathway metabolite and cytokine analysis, while cell lysates were collected only at 48 h for proteomic analyses using a cell scraper.

### 2.3 Cell Imaging and Quantitative Morphological Analysis

C20 and HMC3 cells were seeded in chamber slides (Ibidi, Germany) with a seeding cell density of 10 000 cells /mL. After 24 h, the cell culture medium was replaced with media containing either IFN‐γ (50 ng/mL) (Miltenyi Biotec), or LPS (1 μg/mL). 48 h after treatment, culture media was aspirated, and cells were washed with PBS (Thermo Fisher Scientific, MA, USA). Cells were then fixed in 10% neutral buffered formalin (Sigma‐Aldrich, MO, USA) at room temperature for 20 min. After, formalin was removed, cells were washed with PBS and stained with DAPI (Thermo Fisher Scientific, MA, USA) and AlexaFluor 488 phalloidin (Thermo Fisher Scientific, MA, USA) to visualise the nucleic acids and f‐actin cytoskeleton, respectively. Cells were then incubated in a light‐free environment at room temperature for 20 min. After incubation, staining mixture was removed, replaced with normal PBS, and cells were brought to imaging.

Cells were imaged using an EVOS FL Digital Inverted Fluorescence Microscope (Thermo Fisher Scientific, MA, USA). F‐actin fluorescence recorded at 488 nm excitation and 490–624 nm emission, and DAPI fluorescence was imaged at 405 nm excitation and 410–483 nm emission using GFP (Thermo Fisher Scientific, MA, USA) and DAPI (Thermo Fisher Scientific, MA, USA) LED light cubes. Images (1280 × 960 pixels) were taken using 20× objective.

Quantitative morphological analysis was conducted using ImageJ (Fiji, version 2.14.0). Images were converted to single channel greyscale images (8‐bit) and the signal intensity thresholds were individually adjusted to capture the nuclei and f‐actin. Images were binarized, underwent the erosion operation, and dilation to smooth objects and eliminate isolated pixels. Watershed segmentation was performed to automatically separate adjacent cells, and the ‘Fill holes’ command was employed to fill the segmented cell's background. Measurements included cell area and cell perimeter (based on f‐actin‐positive regions), and the cell shape descriptors (circularity, and perimeter to area ratios), as well as the area and circularity for the nuclei (based on DAPI‐positive regions). The circularity is a ratio of the shortest and longest dimensions of the structure. The circularity equal 1 indicates a perfect round structure. The perimeter to area ration was used a measure of the cell surface roughness, or the extent of ramification. In addition, the nuclear‐cytoplasmic ratio (NCR) was calculated as by dividing the segmented area of the nucleus by the segmented area of f‐actin positive cell zone. The rectangular selection tool was used to capture the image's region of interest. Measurements were taken using the ‘Particle Analyser’ command, within the 5 ‐ ∞ pixel^2^ size.

### 2.4 Cell Lysate and Sample Preparation for LC–MS/MS Analysis

The cells were washed thrice with PBS, and whole cell protein extraction was performed with ice‐cold cell lysis buffer (1% sodium deoxycholate in 0.1 M triethylammonium bicarbonate, Sigma‐Aldrich, MO, USA) using a probe sonicator (Branson Soni‐fier 450; ten bursts at 40% amplitude, output 2 setting) [39]. Cells were then heated at 95°C for 5 min before storage at −80°C for further analysis. To determine protein concentration, collected cell lysates were measured using the Pierce BCA protein assay kit (Thermo Fisher Scientific, MA, USA) according to the manufacturing protocol. Protein samples were normalised to the same concentration and reduced with 15 mM dithiothreitol (Sigma‐Aldrich, MO, USA) at 60°C for 30 min followed by alkylation with 30 mM iodoacetamide (Sigma‐Aldrich, MO, USA) for 30 min at room temperature in the dark. Samples were then digested with sequencing grade porcine trypsin (Promega, WI, USA) at a protease to substrate ratio of 1:30 at 37°C for 16 h at 600 rpm. Samples were dried using vacuum centrifugation for approximately 90 min. Samples were subsequently desalted and cleaned with C18 StageTips prior to Liquid Chromatography with Tandem Mass Spectrometry (LC–MS/MS) [40]. For spectral protein/peptide library generation, all samples were pooled prior to desalting and cleaning with Sep‐Pak C18 Cartridges (Waters Corporation, MA, USA), adhering to the manufacturer's protocol. Digested peptide mixtures were further fractionated into 20 fractions using a high pH reversed phased C18 peptide fractionation method on a 1260 high performance liquid chromatography (HPLC) system (Agilent, Santa Clara, CA, USA) as previously described [41].

### 2.5 LC‐MS/MS Analysis

Samples were analysed utilising a Vanquish ultra‐high performance liquid chromatography (UHPLC) system coupled to an Orbitrap Exploris 480 mass spectrometer (Thermo Fisher Scientific, MA, USA). 600 ng of samples were injected onto the peptide trap column and washed with loading buffer (0.1% formic acid in water). The peptide trap was then switched in‐line with the analytical nano‐LC column, which was an in‐house packed ReproSil‐Pur 120 C18‐AQ (3 um, 250 × 0.075 mm, 75 μm × 30 cm). Peptides were eluted from the trap onto the nano‐LC column and separated with a linear gradient of 2.5% mobile phase B to 37.5% mobile phase B over 60 min at a flow rate of 300 nL/min, then held at 95% mobile phase B for 10 min. The column eluent was directed into the ionisation source of the mass spectrometer operating in positive ion mode. 20 high pH library fractions were analysed using a data‐dependent acquisition (DDA) method, and samples for quantitation were analysed using a data‐independent acquisition (DIA) method as described below.

### 2.6 DDA Spectral Library Generation

Protein spectral library utilising the 20 fractionated peptides were generated independently. The column eluent was directed into the ionisation source of the mass spectrometer operating in positive ion mode. Survey scans from 350 to 1450 m/z at 60 K resolution were recorded at 60 K resolution at 200 m/z in the orbitrap analyser. Precursor ions with a minimum value of 5 × 10^3^, +2 to +6 charge states, were subjected to MS/MS analysis with a fixed cycle time of 1.5 s. The precursor survey scans were fragmented by higher‐energy collisional dissociation (HCD) using a normalised collision energy of 27, 15 K resolution at 200 m/z. Dynamic exclusion was enabled, with a 15 s exclusion duration ±10 ppm.

### 2.7 DIA

The column eluent was directed into the ionisation source of the mass spectrometer operating in positive ion mode. A MS1 scan was performed from 350 to 1450 m/z at 60 K resolution, followed by MS2 scans at 20 m/z ranges and fragmented by HCD using a normalised collision energy of 27. The MS2 scans had scan resolution was set at 30 K. The Automatic Gain Control target and maximum injection time mode parameters were set to “Standard” and “Auto”, respectively.

### 2.8 Data Processing

High pH library fractions DDA data was utilised to generate an ion library using MSFragger (version 3.8) in the Fragpipe GUI (version 20.0) [42]. DDA files were searched against a database of human proteins (Uniprot proteome UP000005640, downloaded June 2023, containing 82 492 protein sequences with all sequences reversed as decoy targets) in the default workflow, with trypsin digestion, peptide length set to 9–30, peptide mass range set to 500–5000 and max missed cleavages set to 2. Peak matching precursor and fragment mass tolerance were set to 20 ppm. While Oxidation of Met and acetylation of N‐terminal were set as variable modifications and carbamidomethylation of Cys was set as a fixed modification, with maximum variable modifications on a peptide set to 3. The spectral library of b and y ions was generated from the search results using SpecLib, with automatic selection for retention time calibration, RT Lowess fraction set to 0.01, Unimod tolerance set to 0.02 Da and fragment tolerance set to 15 ppm.

DIA‐NN (version 1.8) based on the library generated above was used to quantify proteins using a double‐pass neural network classifier [43]. MS1 accuracy, Mass accuracy and scan window were set to 0 for automatic determination, match between runs options was on and the “Heuristic protein inference” option was also on. Precursor false discovery rate (FDR) was set at 1%, protein inference was performed at a Gene level, with a high accuracy LC quantification strategy, RT‐dependent cross‐run normalisation. Subsequently, all diagrams were generated in R using the proteins meeting the specified criteria from the ANOVA analysis, providing a visual representation of the observed patterns and trends.

### 2.9 Ingenuity Pathway Analysis (IPA) and Gene Ontology (GO) Gene Set Enrichment Analysis

IPA (Ingenuity Systems/Qiagen, Redwood City, CA, USA) was used to associate significant protein lists (FDR < 0.05) with biological pathways. The canonical and functional pathway analysis was employed to identify significant biological functions and pathways. Biological functions and pathways were considered statistically significant when z‐score ± 2, and *p*‐value < 0.05, determined by Fisher's exact test.

GO gene set enrichment analysis was conducted using the R package ‘ClusterProfiler’ [44] to compare differentially expressed protein clusters by their enriched biological processes. Background GO set from the Human Molecular Signatures Database were selected for the analysis, and only pathways with three or more enriched genes were included. A strict cutoff was applied with corrected (Benjamini‐Hochberg) *p*‐values < 0.05 and *q*‐value < 2.

### 2.10 Quantification of the Kynurenine Pathway Metabolites

A total of 150 μL of collected cellular supernatant was deproteinized using 10% (w/v) trichloroacetic acid (Sigma‐Aldrich, MO, USA) of an equal volume and incubated for 5 min. Next, deproteinized supernatant was filtered through 0.22 μm syringe filters (Millex, Merck), as previously described [45]. Tryptophan, kynurenine, 3‐hydroxykynurenine, 3‐hydroxyanthranilic acid, and anthranilic acid were analysed simultaneously using an Agilent 1290 UHPLC system. 10 μL of the filtered extract was injected into the UHPLC analyser. The kynurenine pathway metabolites were separated under 40°C for 12 min. The mobile phase was 0.1 mM sodium acetate (pH 4.6, Sigma‐Aldrich, MO, USA) and the separation was carried out in an isocratic flow rate of 0.75 mL/min in an Eclipse Plus C18 reverse‐phase column (2.1 × 150 mm, 1.8 μm particle size; Agilent Technologies Inc., Santa Clara, CA). 3‐hydroxykynurenine and kynurenine were detected using UV detector set at wavelength of 365 nM, while tryptophan, 3‐hydroxyanthranilic acid and anthranilic acid were measured using fluorescence intensity set at Ex/Em wavelength of 280/438 for tryptophan and 320/438 for 3‐hydroxyanthranilic acid and anthranilic acid. Kynurenic acid quantifications were carried out using a reverse phase C18 column (ZORBAX XDB, 4.6 × 100 mm; Agilent Technologies Inc) in an Agilent HPLC system. The mobile phase (50 mM sodium acetate, with 50 mM zinc and 5% v/v HPLC grade acetonitrile, pH 5.2) was run at an isocratic flow rate of 1 mL/min under 35°C for 8 min. Kynurenic acid was detected using fluorescence intensity set at Ex/Em wavelength of 344/388. A series of standards for all metabolites were used for a six‐point calibration curve to interpolate the quantity of the sample readout. Agilent OpenLAB CDS ChemStation (Edition C.01.04) was used to analyse the chromatogram. The inter‐ and intra‐assay coefficient of variation was set at within the acceptable range of 3%–7%.

### 2.11 Measurement of Cytokine Production

The collected culture supernatants were prepared in the 96‐well plate and were centrifuged at 1500 rpm for 5 min and transferred onto a new 96‐well plate. Next, cytokine release was measured using a bead‐based multiplex kit on a human M1/M2 macrophage panel (Biolegend, 740509), following the manufacturer's instructions, using LSRFortessa (BD Biosciences). The cytokines and chemokines were interleukin (IL)‐ 1β, 1RA, 6, 10, 12p40, 12p70, 23, tumour necrosis factor alpha, C‐C motif chemokine ligand 17, and C‐X‐C motif chemokine ligand 10.

### 2.12 Figure Generation

Figures were generated using heatmap3 [46], ggplot2, ggvenn, and VennDiagram [47] within the R environment (R version 4.3.1).

### 2.13 Statistical Analysis

The obtained quantitative imaging data was analysed statistically to compare HMC3 and C20 morphological features in unstimulated state and to examine the effects of LPS and IFN‐γ stimulation on these cells. The data are expressed as means ± standard deviations, unless otherwise specified. A one‐sample Kolmogorov–Smirnov test was applied to examine the distribution of the values of the studied variables. It showed that the distributions are non‐Gaussian for most of the studied variables. Therefore, the non‐parametric methods were used for the intergroup comparisons, including the Kruskal‐Wallis test for several independent groups with post hoc pairwise group comparisons and the two‐sided Mann–Whitney *U*‐test (for comparisons of two independent groups). Statistical analyses were performed using SPSS 29.0.0 (Microsoft, USA) and GraphPad Prism 10.1.0 (GraphPad Software, USA) software.

The proteomic dataset underwent a filtering process, retaining protein groups with observations in a minimum of two replicates per group/treatment within the cohort to ensure data robustness. Subsequently, data normalisation was applied, aligning the median peak intensity of each sample with the global median. In cases of missing data within a group, imputation was carried out by selecting random values from the population's average standard deviation of genes around the minimum observed intensity value. For imputing undetected data within an entire treatment group, values were selected based on the lowest value across the dataset for each cell line (C20: 35543.5; HMC3: 27560.8).

To assess the relative abundance of proteins between the groups, a statistical analysis was conducted using a *t*‐test within the *R* statistical environment. ANOVA *p*‐value was adjusted for multiple testing by Benjamini‐Hochberg for FDR adjustment. Proteins meeting the criteria of statistical significance, defined as having a *p*‐value < 0.05 and a ± 2‐fold change (FC), were considered differentially expressed in the cell line comparison.

The kynurenine pathway metabolite and cytokines were analysed using two‐way ANOVA corrected with Turkey's multiple comparisons test. The differences between the compared groups of values were statistically significant at a *p*‐value < 0.05. Graphs were created using GraphPad Prism 10 (GraphPad, San Diego, USA).

## 3 Results

### 3.1 Morphological Analysis Revealed Distinct Structural and Functional Characteristics of HMC3 and C20 Cells

**FIGURE 1:**
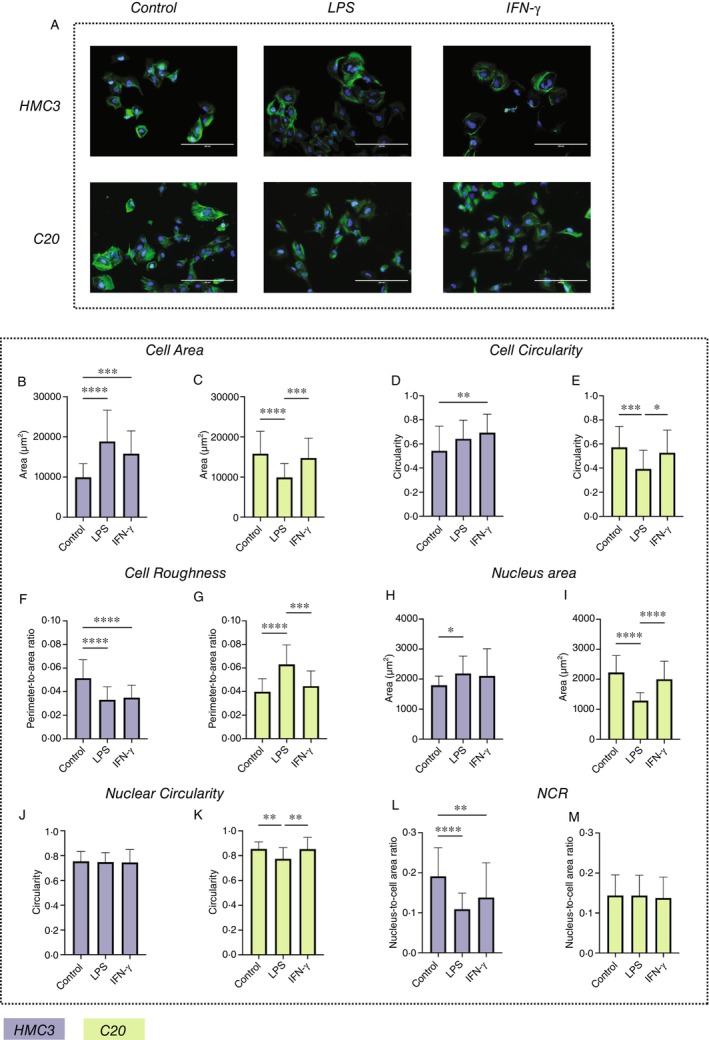
(A) Morphological features of HMC3 and C20 cells. Epifluorescence microscopy, staining with DAPI (blue) for cell nuclei and AlexaFluor 488 phalloidin (green, f‐Actin cytoskeleton). Scale bar 200 μm. The contrast of the images was enhanced for presentation purposes. Note a smaller size and reduced density of f‐Actin in perinuclear region in untreated HMC3 cells, compared with C20 cell line, which demonstrates a more even and dense distribution of f‐Actin filaments across the whole cell body. This feature of f‐Actin intracellular distribution is unaffected by LPS and IFN‐γ stimulation. However, the size and shape parameters of HMC3 and C20 cells are changing in different ways. (B–M) Effect of LPS and IFN‐γ treatment on the morphological characteristics of HMC3 and C20 cells.

The control (untreated) HMC3 and C20 cells showed distinct morphological profiles. HMC3 cells demonstrated balanced polygonal shapes with centrally located ellipsoid nuclei. F‐actin staining in HMC3 cells was enhanced at the cells' periphery and reduced in the perinuclear areas, while C20 cells had a higher density of f‐actin filaments distributed throughout the cytoplasm, with no apparent concentration along the cell contours (Figure 1A).

Compared with HMC3 cells, untreated C20 cells showed a larger size of the cells (*p* < 0.001, Figure 1B,C) and a similar cell circularity (*p* = 0.399, Figure 1D,E). At the same time, the cell surface of HMC3 cells was rougher than in C20 cells (*p* < 0.001, Figure 1F,G). Untreated C20 cells showed larger nuclei (*p* = 0.008, Figure 1H,I), while the shape of the nuclei in C20 cells was significantly rounder than in HMC3 cells (*p* < 0.001, Figure 1J,K). The NCR was statistically significantly higher in HMC3 than in C20 cells (*p* = 0.002, Figure 1L,M).

LPS treatment induced essentially opposite responses in HMC3 and C20 cells, compared to their untreated matching controls (Table S1). In HMC3 cells, exposure to LPS resulted in an approximately 2‐times increase in cell size (*p* < 0.0001, Figure 1B) and slight growth of the nuclear areas (*p* = 0.011, Figure 1H), reduction in the cell surface roughness (*p* < 0.0001, Figure 1F) and NCR (*p* < 0.0001, Figure 1L). The cell and nuclear circularity remained unchanged (Figure 1A,D,J). In contrast, in C20 cells, the f‐actin positive area (cell size) decreased, compared to the control (*p* < 0.0001, Figure 1C), the cell shape became more elongated as reflected by diminished cell circularity (*p* < 0.001, Figure 1E). In parallel, the perimeter to cell area ratio increased, indicating the formation of cytoplasmic protrusions and a more rougher cell surface (*p* < 0.0001, Figure 1G). The nuclei of C20 cells under LPS treatment shrink (*p* < 0.0001, Figure 1I) and become more elongated (*p* = 0.01, Figure 1K) than in control. At the same time, NCR of C20 cells remain unaffected by LPS (Figure 1M).

IFN‐γ treatment did not affect the morphology of C20 cells (Figure 1A), in comparison with control. In HMC3 cells, the trends in morphological modifications induced by IFN‐γ were similar to those observed under LPS treatment. The size of the cells and cell circularity were increased (*p* < 0.001 and *p* = 0.005, respectively, Figure 1B,D), while cell surface roughness decreased (*p* < 0.0001, Figure 1F). There were no statistically significant changes of the size and the circularity of the nuclei of HMC3 cells following IFN‐γ exposure (Figure 1H,J). However, the NCR of HMC3 cells decreased, compared to control (*p* = 0.01, Figure 1L). In addition, under both LPS and IFN‐γ stimulation, HMC3 formed few giant multinucleated cells (Figure 1A). This was a relatively rare observation, slightly more notable in the IFN‐γ treated group.

### 3.2 Comparative Proteomic Analysis of Unstimulated HMC3 and C20 Cells

**FIGURE 2:**
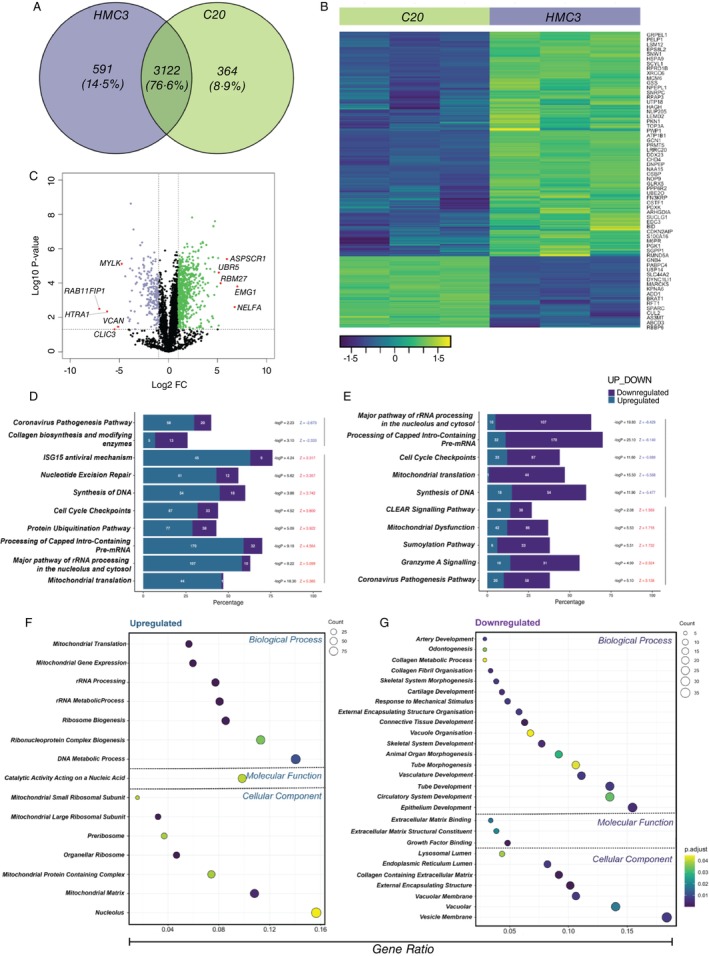
Untreated HMC3 and C20 microglial cells (A) Venn diagram of the shared and unique proteins (found in at least two biological replicates) of both cell lines, (B) Heatmap of all 802 DEPs between HMC3 in comparison to C20, with examples of some DEPs highlighted. 802 DEPs (609 upregulated, 193 downregulated in HMC3), (C) Volcano plot comparing 3122 proteins commonly expressed between HMC3 and C20 cells, with the top 5 down‐ and up‐regulated proteins in HMC3 highlighted in red. The x‐axis represents the fold change of protein expression. Proteins significantly upregulated (green, FC ratio > 2) and downregulated (lilac, FC ratio > −2) in HMC3 relative to C20. The y‐axis represents the −log10 *p*‐value (*p* < 0.05). 802 DEPs (609 upregulated, 193 downregulated in HMC3), (D, E) Bar plot illustrating the results of IPA analysis, highlighting (D) the top 8 and bottom 2 pathways of HMC3 in relation to C20, and (E) the top 5 and bottom 5 pathways of C20 in relation to HMC3 with the highest and lowest z‐score impacted by the DEPs. Bars are colour‐coded to indicate the regulation status of the genes within each pathway. The total number of genes involved in each up/down regulated pathway is annotated in the middle of the respective bars. The bar height reflects the percentage of DEPs implicated in each pathway, providing a visual overview of the significant pathways affected by DEPs in human microglia cells (z‐score + 2, *p* < 0.05), and (F, G) Dot pots illustrating pathway enrichment analysis of (F) up‐, and (G) down‐ regulated DEPs between HMC3 and C20, representing GO terms belonging to one of the three root GO ontologies: Biological process (BP), cellular component (CC), or molecular function (MF) respectively.

To understand the molecular characteristics of HMC3 and C20 cells under normal physiological condition, we carried out proteomic profiling of these cells with no treatment. We detected 3713 proteins in HMC3 and 3486 proteins in C20 cell lines. Of these proteins, we found 591 (14.5%) and 364 (8.9%) unique proteins in HMC3 and C20, respectively, while 3122 proteins (76.6%) were shared between both the cell lines (Figure 2A; Table S2). Among the 3122 proteins detected in both cell lines. 802 (25.7%) proteins were differentially expressed between HMC3 versus C20 cells, including 609 upregulated and 193 downregulated proteins (Figure 2B; Table S2).

The top upregulated differentially expressed proteins (DEPs) in HMC3 cells (downregulated in C20) was EMG1 N1‐Specific Pseudouridine Methyltransferase (EMG1) exhibiting the most substantial increase, with a FC of 129.4 and a *p*‐value of 0.0001. In contrast, the significantly downregulated DEPs in HMC3 (upregulated in C20) was RAB11 Family Interacting Protein 1 (RAB11FIP1) showed prominent down‐regulation, with a remarkable FC of 142.9 and a *p*‐value of 0.003 (Figure 2C). Interestingly, the top‐upregulated DEPs in HMC3 and C20 were associated with transcriptional activity and cytoskeleton organisation, respectively, and these associations were reflected in the canonical pathway analysis and pathway enrichment analysis below.

The IPA analysis of canonical pathways highlighted key differences between HMC3 and C20 microglial cells. HMC3 cells showed activation in pathways related to mitochondrial function, protein synthesis, cell proliferation, DNA repair, and antiviral responses. In contrast, inhibited pathways in HMC3 indicate an indirect role in regulating collagen in the extracellular matrix [48] (Figure 2D). For C20 cells, the most upregulated pathways involved lysosomal activity, responses associated with immune challenge, and mitochondrial stress. The downregulated pathways in C20 were associated with transcription activity (Figure 2E). These differences were further supported by pathway enrichment analysis results, which show that HMC3 cells emphasise activities that are associated with translation and mitochondrial function, while C20 cells demonstrate a reduced capacity for and a focus on cellular structural processes (Figure 2F,G).

### 3.3 Low Percentage of DEPs in Microglia Cell Lines Is Involved in Innate Immune Response

Next, we sought to investigate whether the proteins identified in both cell lines are associated with innate immunity‐related activities. To achieve this, we first obtained a curated list of GO terms specifically related to innate immunity response (GO: 0045087) from the GO Consortium. Subsequently, we compared this list of innate immunity‐related genes against the protein lists obtained from our proteomics data for both HMC3 and C20 cell lines.

**FIGURE 3:**
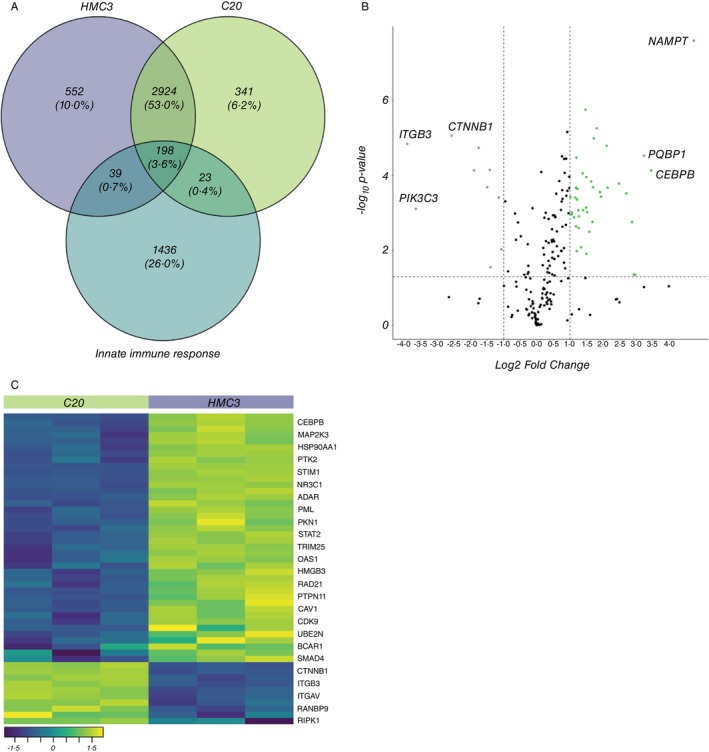
(A) Venn diagram showing overlapping and unique altered proteins associated to the innate immunity response (GO:0045087), HMC3, and C20, (B) Volcano plot comparing 198 common proteins between the innate immunity response (GO:0045087), HMC3, and C20. Of which, 50 proteins were differentially expressed (10 downregulated, and 40 upregulated) in HMC3 compared to C20 (FC ratio + 2, *p* < 0.05), (C) Heatmap of differentially expressed common proteins between HMC3, C20 and the innate immunity response (GO:0045087). A total of 50 proteins were differentially expressed, with some examples highlighted: 10 proteins were downregulated, and 40 proteins were upregulated in HMC3 (indicating that these 10 proteins were upregulated, and the 40 proteins were downregulated in C20) (FC ratio + 2, *p* < 0.05).

Our analysis revealed that, among the genes identified in HMC3, only 237 (4.3%) genes were associated with innate immunity, while C20 had 221 (4%) genes associated with innate immunity. Notably, our comparison of these protein lists revealed a significant overlap, with 198 genes (3.6%) being shared between innate immunity‐related genes and both HMC3 and C20 cell lines (Figure 3A). Of these 198 commonly shared proteins, 40 DEPs were upregulated and 10 were downregulated in HMC3 when compared to C20 (Figure 3B,C).

The most upregulated DEP in HMC3, and consequently downregulated in C20, was Nicotinamide Phosphoribosyltransferase (NAMPT), while the most downregulated DEP in HMC3 (upregulated in C20) is Integrin Beta 3 (ITGB3) (Figure 3B,C). Interestingly, our comparative analysis of HMC3, C20 and GO:0045087 revealed a distinct subset of 1436 proteins (26% of total genes; Figure 3A) that were exclusively associated with the innate immune response. This high proportion of proteins unrelated to either HMC3 or C20 could potentially be due to the absence of an immune challenge or their presence at low, undetectable levels in both cell lines.

### 3.4 Low Value Imputation Reveals Treatment‐Dependent Protein Expression Changes

To understand immune response capacity of each microglia cell line, we challenged them with LPS, or IFN‐γ. Our proteomic analysis revealed DEPs between control and treatment groups, including proteins undetectable in either group. The emergence or loss of these proteins suggests treatment‐amplified or reduced expression, where the applied treatment either induces or decreases the expression of these proteins to detectable or undetectable levels, respectively.

This indicates a potent regulatory effect of the treatment on the proteomic landscape. This effect on protein expression may have significant biological implications for affected cellular processes and pathways. Protein groups that had been observed in all triplicates in one group but were not detected in the other were manually imputed with the lowest value in the entire dataset for that cell line prior to statistical analysis (Table S4).

**FIGURE 4:**
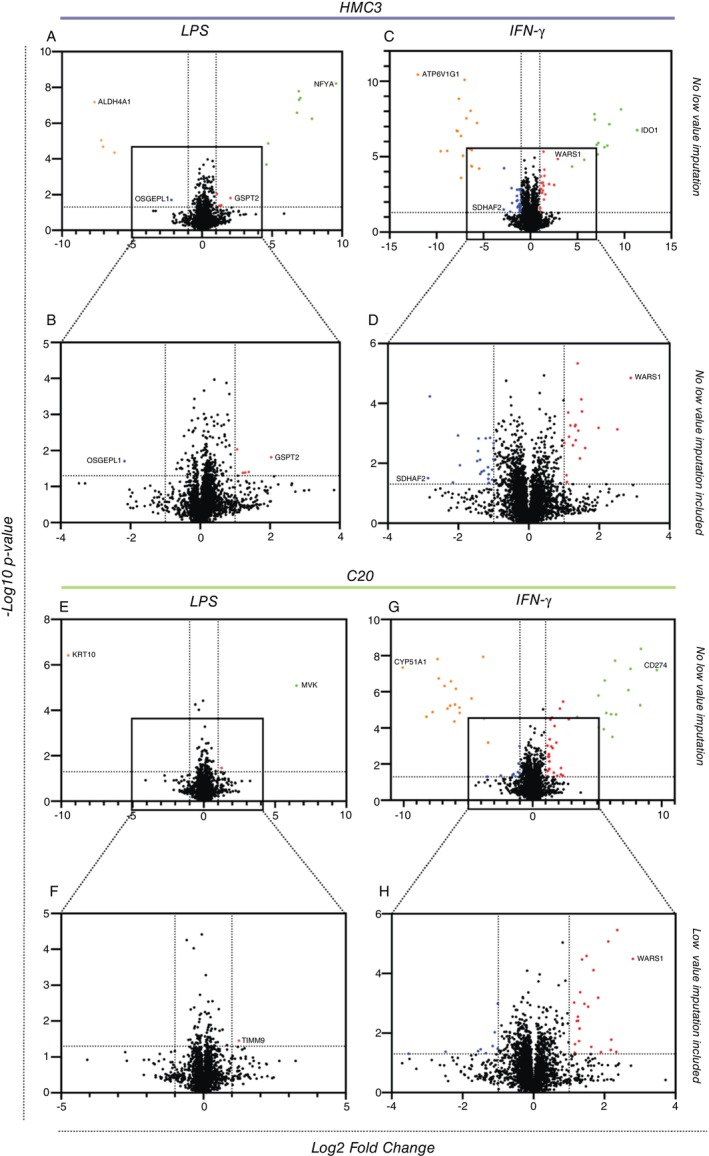
Cell protein quantification in LPS and IFN‐γ treated groups in (A–D) HMC3 and (E–H) C20. Volcano plot representations on DEPs (FC ratio + 2, *p* < 0.05) between control and treatment groups. Red dots indicate upregulated proteins, green dots indicate upregulated low value imputed proteins, blue dots indicate down‐regulated proteins, and orange dots indicate down‐regulated low value imputed proteins. Dotted lines on the X‐axis are log2 FC = −1 and 1.

In HMC3 cells, 11 and 32 proteins were differentially expressed between the control and LPS (Figure 4A) or IFN‐γ (Figure 4C) treatment groups, respectively, while in C20 cells, 2 and 33 proteins were differentially expressed between the control and LPS (Figure 4E) or IFN‐γ (Figure 4G) when manually imputed.

In LPS‐treated HMC3 cells, Nuclear Transcription Factor Y Subunit Alpha (NFYA) was the most significantly upregulated protein, while Aldehyde Dehydrogenase 4 Family Member A1 (ALDH4A1) was the most significantly downregulated protein when compared against control group (Figure 4A). IFN‐γ treated HMC3 cells showed that Indoleamine 2,3‐dioxygenase‐1 (IDO1) was the most significantly upregulated protein, while ATPase H+ Transporting V1 Subunit G1 (ATP6V1G1) was the most significantly downregulated protein compared to the control group (Figure 4C).

In C20, Mevalonate Kinase (MVK) was the most significantly upregulated protein, while Keratin 10 (KRT10) was the most significantly downregulated protein when compared against control and LPS treated group (Figure 4E). CD274 was the most significantly upregulated protein, while Cytochrome P450 Family 51 Subfamily A Member 1 (CYP51A1) was the most significantly downregulated protein when compared against control and IFN‐γ treated group (Figure 4G).

### 3.5 Proteomic Analysis of DEPs Revealed Distinct Proteomic Profiles in Immune Challenged HMC3 and C20

A total of 47 and 16 proteins were differentially expressed in HMC3 and C20 cell lysates, respectively, across LPS and IFN‐γ treatment groups. To better characterise both these microglia cells lines, we investigated the proteomic signatures associated with the treated cell lysates.

In HMC3 cells, a total of 3976 proteins appeared as quantifiable across all samples (Table S5). There were six and 41 DEPs between the control and LPS (Figure 4B) or IFN‐γ (Figure 4D) treatment groups, respectively. Among these six DEPs between control and LPS (Figure 4B), five proteins were upregulated, G1 To S Phase Transition 2 (GSPT2) was the most significantly upregulated protein (FC of 4.1 and *p* < 0.05) while O‐Sialoglycoprotein Endopeptidase Like 1 (OSGEPL1) was the most significantly downregulated protein (FC of 5 and a *p* < 0.05). Increased expression of GSPT2 [49] and Nitrilase 1 (NIT1) [50] suggests that the treatment of LPS restricted the cell proliferation of HMC3 and skewed its biological activity towards antiviral as evidenced by Interferon Stimulated Exonuclease Gene 20 (ISG20), an antiviral gene [51]. The switch in biological activity could be supported by lipid metabolism, driven by the expression of other DEPs [52, 53], instead of mitochondrial respiration, as indicated by the significant reduction of OSGEPL1 [54].

Among the 41 DEPs were identified in IFN‐γ treated HMC3 cells (Figure 4D), 19 were upregulated, notably Tryptophanyl‐TRNA Synthetase 1 (WARS1) with FC 7.4 (*p* < 0.0001), linked to immune response [55]. Conversely, 22 DEPs were downregulated, with Succinate Dehydrogenase Complex Assembly Factor 2 (SDHAF2) showing the greatest decrease (FC 7.1, *p* < 0.05). Overall, these changes suggest that HMC3 cells shift their metabolic activity to support an antiviral response following IFN‐γ treatment.

In C20 cells, a total of 3698 proteins were quantifiable across all samples (Table S6). One and 15 proteins were differentially expressed between the control and LPS or IFN‐γ treatment groups, respectively (Figure 4F,H). The one DEP between control and LPS was upregulated, and it was Translocase Of Inner Mitochondrial Membrane 9 (TIMM9) (FC of 90.92 and a *p* < 0.0001) (Figure 4F). TIMM9 is one out of two mitochondrial proteins necessary in forming the TIM10 complex that functions as chaperone [56].

In the IFN‐γ group, 15 DEPs were found; six were upregulated, and nine were downregulated. WARS1 was the most upregulated (FC 6.95, *p* < 0.0001), while KRT10 was the most downregulated (FC 11.1, *p* < 0.05) (Figure 4H).

The upregulated proteins highlight their role in enhancing responses to inflammation and infection [57, 58, 59, 60, 61]. Although KRT10's downregulation in microglia may not have a clear effect due to its usual association with epithelial cells [62], it could impact structural stability and resilience, requiring further study. The remaining downregulated DEPs were found to be involved in sodium ion balance [63], RNA processing [64], cell survival [65], and apoptosis [66].

**FIGURE 5:**
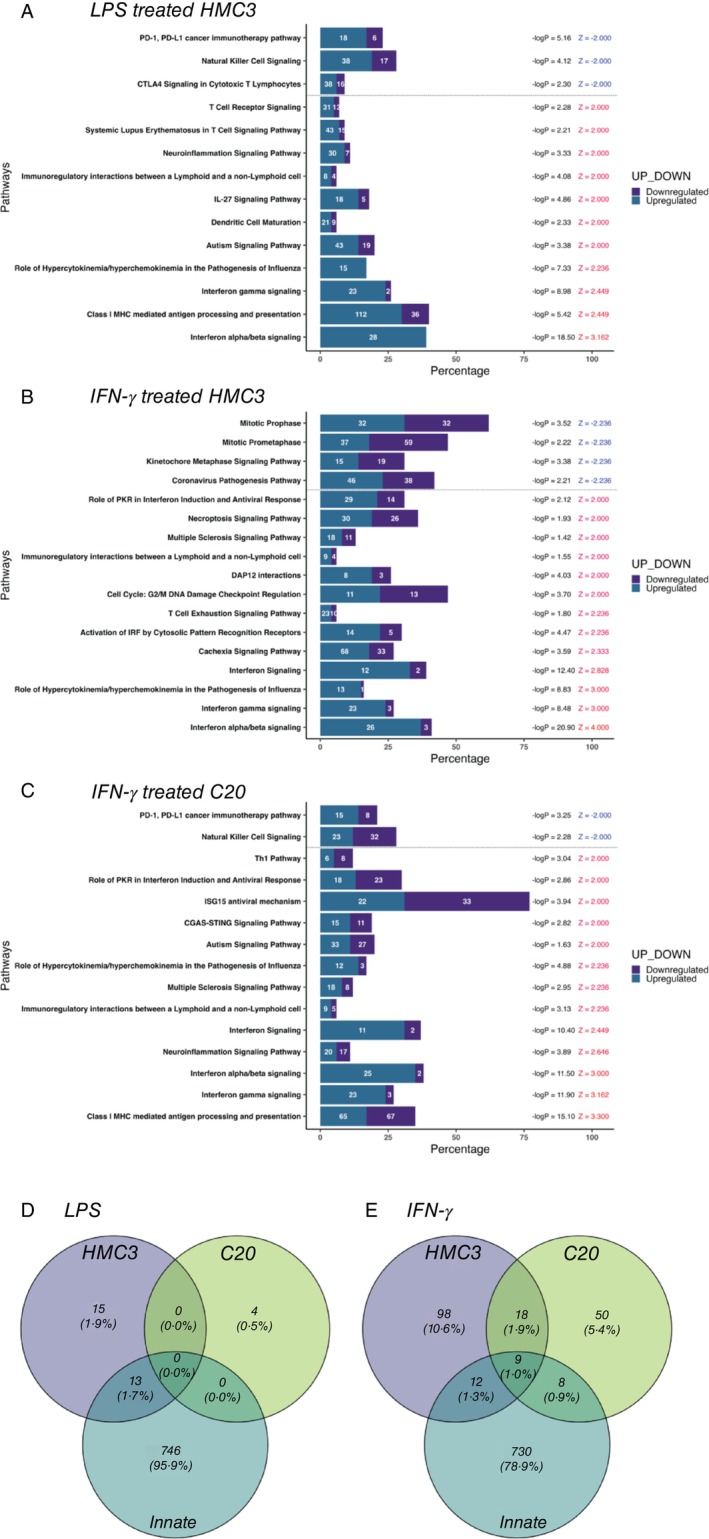
(A–C) Bar plot illustrating results of IPA analysis, identifying cellular pathways impacted by the differential expression of proteins (A) LPS treated HMC3, (B) IFN‐γ treated HMC3, and (C) IFN‐γ treated C20 (z‐score > 2 or < −2, −log10 *p* < 1.3), and (D, E) Venn Diagram displaying common and unique DEPs associated to the innate immunity response (GO:0045087), HMC3, and C20 when challenged with (D) LPS, or (E) IFN‐γ.

IPA analysis identified key pathways impacted by DEPs following LPS and IFN‐γ treatment in both microglial cells. HMC3 cells treated with LPS, showed DEPs involved in 14 immune response related pathways (Figure 5A), while IFN‐γ treatment impacted 17 pathways, mainly linked to DNA synthesis and immune response (Figure 5B). Alternatively, IFN‐γ treated C20 cells engaged 15 unique immune response pathways (Figure 5C), indicating that the two cell lines respond differently to IFN‐γ. Notably, C20 did not show LPS‐related pathway activation, suggesting a specific response tied to cell type.

Despite identifying immune‐associated pathways, only a small proportion of DEPs were linked to innate immunity (GO:0045087) (Figure 5D,E). LPS‐treated C20 cells showed no association with innate immunity DEPs, whereas 13 proteins (1.7%) in LPS‐treated HMC3 were linked to innate immune functions, associated with antiviral defence and inflammation [67]. IFN‐γ treatment showed nine (1%) DEPs common across HMC3, C20, and GO:0045087, involved in antigen presentation and antiviral responses [68, 69, 70, 71, 72]. Additionally, HMC3 had 12 DEPs (1.3%) enriched in antiviral and immune pathways [51, 72, 73, 74, 75, 76, 77, 78, 79, 80], while C20 had eight DEPs (0.9%) associated with processes like autophagy, apoptosis, and protein regulation (Table S3) [59, 60, 61, 81, 82, 83, 84, 85].

### 3.6 IFN‐γ Significantly Activates the Kynurenine Pathway in HMC3 and C20 Cells

Given the strong association of interferon signalling pathways in IPA analysis of both LPS and IFN‐γ treated HMC3 and C20 cells [86] (Figure 5A,B) and that primary human microglia have been previously shown to produce neurotoxic and/or neuroprotective kynurenine pathway metabolites [87], we next examined the activity of the kynurenine pathway in these cell lines under similar conditions.

**FIGURE 6:**
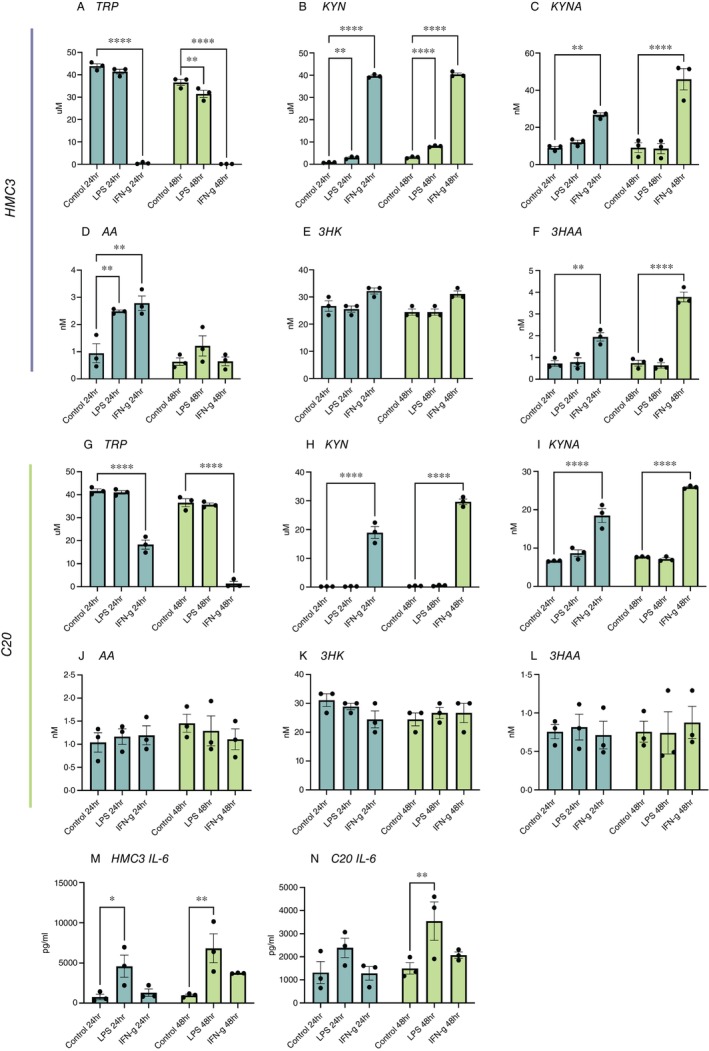
Regulation of the kynurenine pathway (A–F) HMC3 (G–L) C20, and (M, N) Effect of LPS and IFN‐γ treatments on the concentration of IL‐6 in the secretome of (M) HMC3 and (N) C20 cells. Effect of LPS and IFN‐γ treatments on the concentration of IL‐6 in the secretome of HMC3 and C20 cells.

In HMC3 cells, only IFN‐γ treatment significantly activated the kynurenine pathway as tryptophan (24 and 48 h, *p* < 0.0001; Figure 6A) was significantly depleted to enhance kynurenine production (24 and 48 h, *p* < 0.0001; Figure 6B), compared to untreated cells. After that, kynurenine was subsequently catabolised to kynurenic acid (24 h, *p* = 0.01; 48 h, *p* < 0.0001; Figure 6C), anthranilic acid (24 h, *p* = 0.01; Figure 6D) and 3‐hydroxylanthranilic acid (24 h, *p* < 0.01; 48 h, *p* < 0.0001; Figure 6E). The level of 3‐hydroxylkynurenine was higher but not significant in treated cells at both time points and this is likely be due to active catabolism of 3‐hydroxylkynurenine to produce 3‐hydroxylanthrnailic acid (Figure 6F).

There was no activation of the kynurenine pathway in HMC3 treated with LPS as reflected in minimal changes to the level of tryptophan except at 48 h (*p* < 0.01, Figure 6A). Interestingly, the level of kynurenine was significantly depleted in the media at 24 h (*p* = 0.01) and 48 h (*p* < 0.0001) (Figure 6B) to produce anthranilic acid at 24 h (*p* = 0.01; Figure 6D). LPS treatment did not induce any significant changes to the production of kynurenine acid, 3‐hydroxylkynurenine, and 3‐hydroxylanthranilic acid (Figure 6C,E,F).

Similar trend was observed in C20 where the kynurenine pathway was activated only in the treatment of IFN‐γ whereby tryptophan (24 and 48 h, *p* < 0.0001, Figure 6G) was catabolised to produce kynurenine (24 and 48 h, *p* < 0.0001, Figure 6H). Interestingly, we observed that the pathway proceeded only towards production of kynurenic acid (24 h, *p* < 0.0001; 48 h, *p* < 0.0001, Figure 6I) but not anthranilic acid (Figure 6J), 3‐hyroxylkynurenine (Figure 6K) and 3‐hydroxylanthranilic acid (Figure 6L). There was no difference in the level of tryptophan and kynurenine pathway metabolites in LPS treated C20.

### 3.7 IL‐6 Was the Predominant Inflammatory Cytokine in LPS Treated HMC3 and C20

Considering that most of the DEPs in the treated microglia cell lines were associated with the inflammation response, we next examined the inflammatory response of these cells.

HMC3 cells treated with LPS showed a significant increase of IL‐6 production in 24 h (*p* = 0.05) and 48 h (*p* = 0.01) (Figure 6M). The C20 showed an increase of IL‐6 secretion at both 24 and 48 h, but it was statistically significant only at the 48 h time point (*p* = 0.01) (Figure 6N).

After IFN‐γ treatment, an increased level of IL‐6 was observed only at 48 h timepoints in both HMC3 and C20 cells, compared with control, but these changes were not statistically significant. There were no significant differences in the other measured cytokines and chemokines in either treatment (Figure S1).

## 4 Discussion

Microglia play a key role in maintaining a homeostatic environment in the brain by eliminating pathogens and damaged cells before promoting tissue restoration [88]. Defining the molecular characteristics of these microglia cell lines will allow appropriate selection for the study of neuroinflammation and/or neurological diseases such as neurodegeneration and cancer. The current study provides the results of the comparative analysis of the proteomic landscape of HMC3 and C20 cells under normal physiological conditions and when challenged with IFN‐γ and LPS in parallel to the evaluation of cytokine and chemokines secreted by these cells and the functional state of the kynurenine pathway.

Our study revealed distinct morphological features between HMC3 and C20 cells under basal conditions. HMC3 cells were smaller and had a rougher surface, more elongated nuclei, and a higher NCR compared to C20 cells (Figure 1A). In contrast to C20 cells, HMC3 exhibited clear morphological changes in response to both LPS and IFN‐γ treatments, while statistically significant modifications in C20 cells were observed only following LPS exposure, with no changes after IFN‐γ treatment. We found that HMC3 cells responded similarly to LPS and IFN‐γ, with increased cell size, reduced surface ramification, and decreased NCR. C20 cells and their nuclei became smaller and more elongated following LPS treatment. Additionally, the surface roughness of C20 cells increased after LPS exposure, while their NCR remained unchanged. These observations were validated through statistical analysis.

In the brain tissue, immune activated and naïve (homeostatic) microglia are traditionally delineated by their morphological differences. The morphotypes of microglial cells depend on their anatomical origin, age, species and many other factors. However, as a rule, the loss of the ramified morphology characteristic of resting microglia, and the acquisition of an amoeboid shape with increased motility, are well‐recognised hallmarks of microglial immune activation [89]. Maintaining the microglial phenotype in vitro, however, is challenging due to the frequent loss of core microglial signature genes in tissue culture [90], which results in a more macrophage‐like morphology.

Our findings in LPS and IFN‐γ treated HMC3 cells align with the observations of LPS‐reactive microglia of mouse brain described by M. Adrian et al. [91] and the changes induced in rat cortical microglia following the traumatic brain injury [92]. According to the cited work [91], cell shape modifications were linked to microtubule remodelling. This rearrangement of microtubules was associated with the formation of centrosomally‐anchored clusters, facilitating cytokine trafficking and release, regulated by cyclin‐dependent kinase 1. The response of C20 cells to LPS in our study did not correspond well with the current understanding of the morphological changes in activated microglia. However, it possibly can be interpreted as a version of the primed microglia phenotype described elsewhere [93] as a state characterised by cells recovering from a previous inflammatory event and unable to exhibit the full spectrum of immune activation reactivity.

Under normal physiological conditions, the DEPs in our proteomics analysis revealed that HMC3 exhibited a transcriptionally and metabolically active profile, whereas C20 displayed a biosynthetic and structural integrity profile. Although both cell lines were immortalised with SV40, the detected profile differences can be attributed to the cells' origins. HMC3 was transformed from foetal microglia [25], which may explain its active transcriptional characteristics, while C20 was transformed from adult microglia [32]. Furthermore, the lower transcriptional activity in C20 could be attributed to the inclusion of the hTERT gene during immortalization [32]. Another potential explanation for these different characteristics could be related to their phagocytotic proficiency. C20 has been shown to migrate at a speed like primary microglia and is capable of phagocytosing dead cells [32]. This can be supported by our observed higher expression of GNB2 in untreated C20, which has been implicated in enhanced chemotaxis cell velocity [94]. HMC3, on the other hand, has been shown to have low phagocytic activity [26]. Phagocytosis is a process highly dependent on cytoskeletal reorganization [95], which aligns with the observed proteomic profile of C20.

Although the cell lines exhibited microglia functionality such as phagocytosis [25, 32], protein expression analysis of these cell lines was shown to have a low proportion of conserved microglia‐enriched genes [96]. We noted that both cell lines shared a low proportion of detected proteins with innate immunity responses (GO: 0045087) (Figure 2B), and this corroborates with a separate transcriptomic study on these cells [96]. The authors noted that the gene profiles of HMC3 and C20 cells varied very differently from those of primary microglia, which could be attributed to the immortalization process, which introduces genetic variability and the absence of an immune challenge. This could also be the explanation for the low proportion of proteins associated with innate immunity responses shared between HMC3 and C20 when challenged with LPS or IFN‐γ (Figure 4D,E). The proportion of DEPs in LPS‐treated and IFN‐γ‐treated HMC3 shared less than 2% and 3% with innate immunity responses, respectively while IFN‐γ‐treated C20 shared less than 1% with innate immunity responses. Despite a low proportion of DEPs being associated with innate immune responses, IPA revealed that most of the pathways were still linked to immune‐related processes, such as interferon signalling pathways and antiviral immune responses. Notably, WARS1 was the most significantly elevated DEP in both IFN‐γ treated cell lines. In a study led by Lee and colleagues, recombinant WARS treatment induced the release of inflammatory cytokines in human cell lines and inhibited viral replication in monocytes [97]. Another significantly elevated DEP in all treatments of HMC3 was ISG20, which exerts its antiviral activity through the degradation of single‐stranded RNA. This RNA degradation activity has been shown to delay the replication of human immunodeficiency virus [98] and enhance resistance to infection by RNA viruses [99]. Altogether, these data suggest potential antiviral mechanisms in immune‐challenged HMC3 and C20.

The results from IPA highlighted distinct differences in the pathway activation profiles of HMC3 and C20 microglial cell lines in response to LPS and IFN‐γ stimulation, suggesting cell line‐specific functional characteristics.

The observed activation of interferon signalling and Class I MHC‐mediated processes in HMC3 cells following LPS stimulation aligns with established molecular mechanisms described in the literature. LPS is known to activate the nuclear factor‐kappa B (NF‐κB) pathway [100], a key transcription factor in innate immunity, which can induce the production of interferons and subsequently activate downstream interferon‐stimulated genes via the Janus kinase‐signal transducer and activator of transcription (JAK–STAT) pathway [101]. The activation of these pathways in HMC3 cells, as identified in our IPA results, highlights their capacity to mount a strong innate immune response involving antiviral defence and enhanced antigen presentation. Notably, C20 cells fail to exhibit any measurable pathway activation in response to LPS, potentially due to reduced expression or activity of LPS receptors such as Toll‐like receptor 4 [102]. This further underscores the specificity of this response in HMC3 cells and emphasises their distinct immune signalling profile.

Under IFN‐γ stimulation, a pro‐inflammatory cytokine that utilises the JAK–STAT pathway to activate downstream signalling, HMC3 cells demonstrate the activation of antiviral pathways as well as pathways associated with systemic inflammation, such as the cachexia signalling pathway [103]. This indicates a broader immune and metabolic involvement in their response. In contrast, C20 cells activate pathways specific to brain inflammation and antigen presentation, including the neuroinflammation signalling pathway [104, 105], reflecting their specialised role in central nervous system immune processes. These findings underscore the distinct functional characteristics of each cell line, with HMC3 exhibiting a more robust response to both bacterial and inflammatory stimuli, whereas C20 shows a more targeted response to central nervous system‐specific inflammation.

Our findings further indicate that the selective activation of the kynurenine pathway by IFN‐γ is consistent with studies identifying interferon signalling as a key regulator of IDO1 activity [106, 107] and its role in subsequent immune modulation. This aligns with IFN‐γ's ability to drive tryptophan metabolism through IDO1, contributing to an immunoregulatory environment. In contrast, LPS primarily exacerbates IL‐6 production, highlighting its distinct mechanism of promoting inflammatory cytokine cascades rather than engaging metabolic pathways like the kynurenine pathway. This divergence underscores the specific roles of IFN‐γ and LPS in microglial immune responses, with IFN‐γ fostering immune modulation through metabolic reprogramming, whilst LPS amplifies acute pro‐inflammatory signalling. Together, these findings provide insight into how microglia tailor their responses to different immune stimuli, with implications for understanding the regulation of inflammation and metabolic pathways in neuroimmune interactions.

These results corroborate evidence from other studies indicating that microglial cells, although capable of mounting robust immune responses, exhibit selective regulatory pathways depending on the type of stimulus [12, 108, 109]. This emphasises the complex interplay between cytokine signalling, antigen presentation, and metabolic pathways in microglia, suggesting potential implications for understanding immune responses in neuroinflammatory and neurodegenerative contexts.

## 5 Limitations

While the study is centred between two microglial cell lines, HMC3 and C20, it is important to note that cell lines do not capture the extensive heterogeneity of microglia found in the human brain. Additionally, although the application of IFN‐γ and LPS as stimulants provides valuable insights into microglial activation pathways, this does not encompass the wide array of stimuli microglia encounter in physiological and pathological conditions. Furthermore, we did not have access to human primary microglia to compare our findings from HMC3 and C20. Finally, the above experiment was conducted in the absence of other glial cells, such as astrocytes. The characteristics and behaviour of these microglia will change when they are challenged in their immediate environment surrounded by other glial cell types. Addressing these limitations in future work will be crucial to developing a more comprehensive and nuanced understanding of microglial cells.

## 6 Conclusion

This research provides a comprehensive analysis of the proteomics profiles of two human microglia cell lines, C20 and HMC3, under normal physiological conditions and when exposed to IFN‐γ or LPS to understand their biological and immune functions. HMC3 and C20 microglial cell lines offer unique advantages for studying various aspects of microglial biology. HMC3 cells are particularly valuable for investigating pro‐inflammatory responses, while C20 cells provide insights into regulatory mechanisms and chronic inflammation. While our research has identified potential key biological pathways activated during immune challenges, further investigations will be required to validate the specific functions of microglia in various human neurological disorders and conditions.

## Author Contributions

Conceptualization, B.G., A.G., and B.H.; writing – original draft preparation, review and editing, B.G, S.K, S.S.C, M.D.V., A.G, S.B.A and B.H.; experimental work and data analysis – B.G, S.K, S.S.C, M.D.V., A.G, S.B.A and B.H.

## Conflicts of Interest

The authors declare no conflicts of interest.

## Supporting information


**Data S1.** Supporting Information.

**Table S2.** Differentially expressed proteins in untreated basal conditions.

**Table S3.** Proteins from innate immunity GO: 0045087.

**Table S4.** Low‐value imputations.

**Table S5.** Differentially expressed proteins in LPS and IFN‐γ treated HMC3.

**Table S6.** Differentially expressed proteins in LPS and IFN‐γ treated C20.

## Data Availability

The data that supports the findings of this study are available in the Supporting Information of this article.
