# Supplementary material for: Comparative Analysis of HMC3 and C20 Microglial Cell Lines Reveals Differential Myeloid Characteristics and Responses to Immune Stimuli
Source: Immunology. 2025 Feb 17;175(1):84–102. doi: 10.1111/imm.13900 (PMC11982601; doi:10.1111/imm.13900)
Supplement: Supplementary file 1 — Data S1. Supporting Information. [file IMM-175-84-s003.docx]

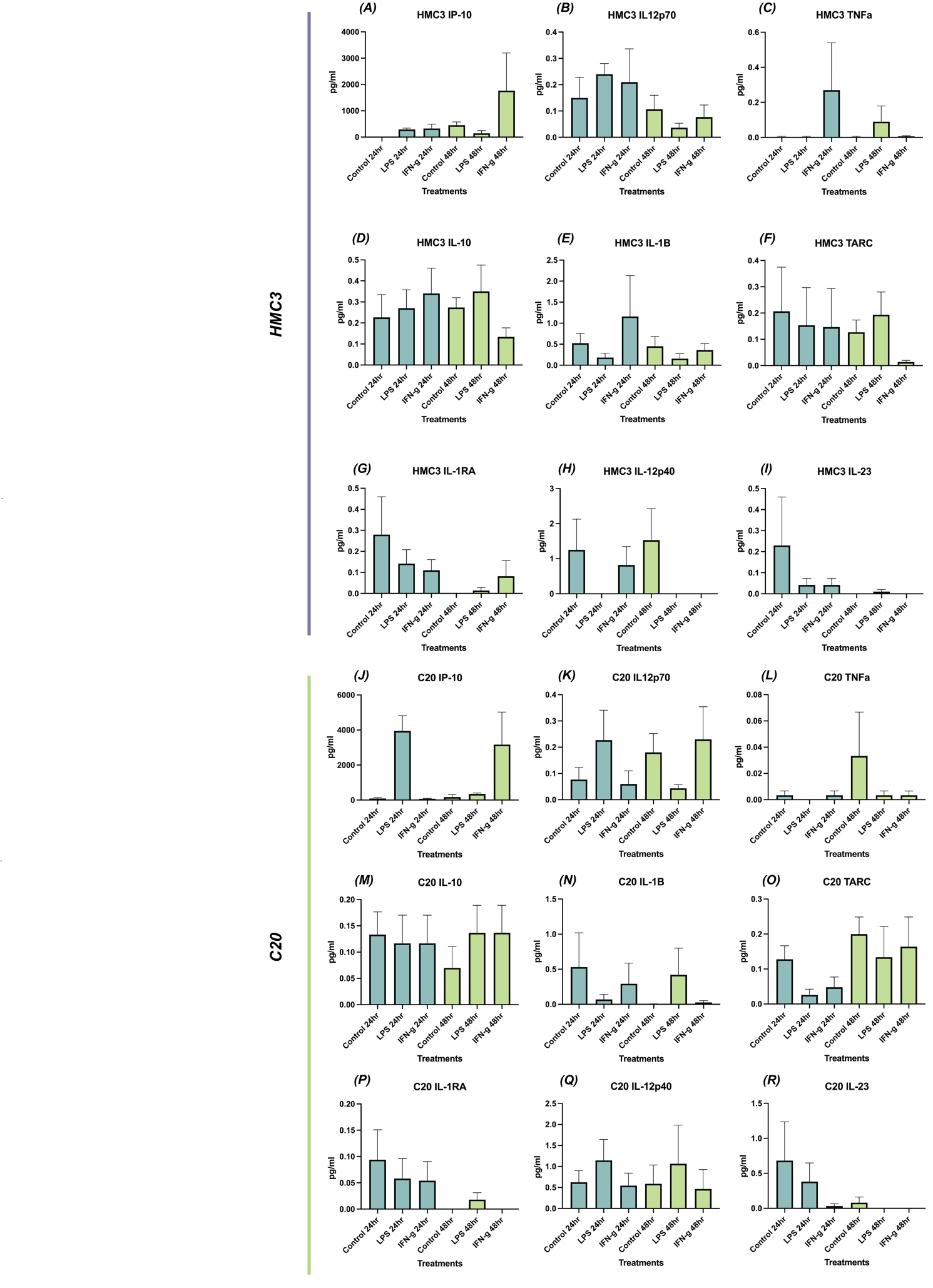


**Supplement Figure 1:** *Effect of LPS and IFN-γ treatments on the concentration of various cytokines and chemokines in the secretome of* ***(A-I)*** *HMC3 and* ***(J-R)*** *C20 cells.*

***Supplementary Tables***

| **Morphological features** | **HMC3** | | | **C20** | | |
| --- | --- | --- | --- | --- | --- | --- |
|  | **Control** | **LPS** | **IFN-γ** | **Control** | **LPS** | **IFN-γ** |
| Cell area, µm^2^ | 10715.20±  5407.21 | 25814.26±  20652.65 | 19643.40±  12810.12 | 17561.63±  8596.45 | 9936.10±  3414.99 | 14733.80±  4972.45 |
| Cell circularity | .54±.21 | .64±.15 | .69±.15 | .57±.17 | .39±.16 | .53±.19 |
| Cell perimeter to area ratio (surface roughness) | .05±.02 | .03±.01 | .03±.01 | .04±.01 | .06±.02 | .05±.01 |
| Area of the cell nucleus, µm^2^ | 1837.27±  410.39 | 2489.53±  1077.12 | 2322.80±  1497.20 | 2222.43±  566.53 | 1313.83±  302.71 | 2119.60±  872.09 |
| Circularity of the cell nucleus | .75±.08 | .75±.08 | .74±.11 | .86±.06 | .78±.09 | .82±.15 |
| NCR, % | .20±.09 | .13±.08 | .15±.11 | .14±.05 | .14±.05 | .16±.09 |

**Supplementary Table 1**. Morphological features of HMC3 and C20 cells under different treatment conditions, Mean ± Standard Deviation.

**Supplementary Table 2**. *Differentially expressed proteins in untreated basal conditions*

**Supplementary Table 3**. *Proteins from innate immunity GO: 0045087*

**Supplementary Table 4**. *Low-value imputations*

**Supplementary Table 5**. *Differentially expressed proteins in LPS and IFN-γ treated HMC3*

**Supplementary Table 6**. *Differentially expressed proteins in LPS and IFN-γ treated C20*
